# Comparative prospects of imaging methods for whole-brain mammalian connectomics

**DOI:** 10.1016/j.crmeth.2025.100988

**Published:** 2025-02-18

**Authors:** Logan Thrasher Collins, Todd Huffman, Randal Koene

**Affiliations:** 1 Washington University in St. Louis, Department of Biomedical Engineering, St. Louis, MO, USA; 2 E11 Bio, Alameda, CA, USA; 3 Carboncopies Foundation, Sacramento, CA, USA

**Keywords:** Connectomics, imaging, expansion microscopy, electron microscopy, light-sheet fluorescence microscopy, brain

## Abstract

Mammalian whole-brain connectomes are a foundational ingredient for a holistic understanding of brains. Indeed, imaging connectomes at sufficient resolution to densely reconstruct cellular morphology and synapses represents a long-standing goal in neuroscience. Mouse connectomes could soon come within reach, while human connectomes remain a more distant yet still worthy goal. Though the technologies needed to reconstruct whole-brain connectomes have not yet reached full maturity, they are advancing rapidly. Close examination of these technologies may help plan connectomics projects. Here, we quantitatively compare imaging technologies that have the potential to enable whole-brain mammalian connectomics. We perform calculations on electron microscopy (EM) techniques and expansion light-sheet fluorescence microscopy (ExLSFM) methods. We consider techniques that have sufficient resolution to identify all synapses and sufficient speed to be relevant for whole mammalian brains. We offer this analysis as a resource for those considering how to organize efforts toward imaging whole-brain mammalian connectomes.

## Teaser

Collins et al. compare the potential of leading imaging technologies (electron microscopy and expansion light-sheet fluorescence microscopy) for connectomics projects aimed at mapping entire mammalian brains with nanoscale resolution, quantitatively examining metrics like speed, voxel size, and cost to evaluate the benefits and drawbacks of the most promising microscopy methods.

## Introduction

Connectomics at nanoscale resolution presents a major technical challenge due to the tremendous amounts of image data involved.^1^^,^^2^ To reach sufficient resolution for accurately reconstructing fine neurites and synapses, voxel dimensions on the order of just a few tens of nanometers or less are needed. But with the recent completion of the larval *Drosophila* brain connectome,^3^ adult *Drosophila* brain connectome,^4^^,^^5^ adult *Drosophila* ventral nerve cord connectome,^6^^,^^7^ and larval *Platynereis dumerilii* connectome,^8^ as well as the less recent completion of the first *C. elegans* connectome,^9^ it seems that the age of whole-brain connectomics has arrived. Projects with the eventual goal of mapping a whole-brain mouse connectome have recently launched, which has prompted discussions about the eventual goal of human brain connectomics. However, the *Drosophila* brain volume is only 0.0175 mm^3^, while the mouse brain has a volume of around 500 mm^3^, and the human brain has a volume in the range of 1,200,000 mm^3^.^2^^,^^10^^,^^11^ That is, the mouse brain has a volume of about 28,571 times that of the *Drosophila* brain, while the human brain has a volume of about 2,400 times greater still compared with the mouse brain. Dramatic increases in imaging throughput will be necessary for whole-brain mammalian connectomics endeavors.

**Figure 1:**
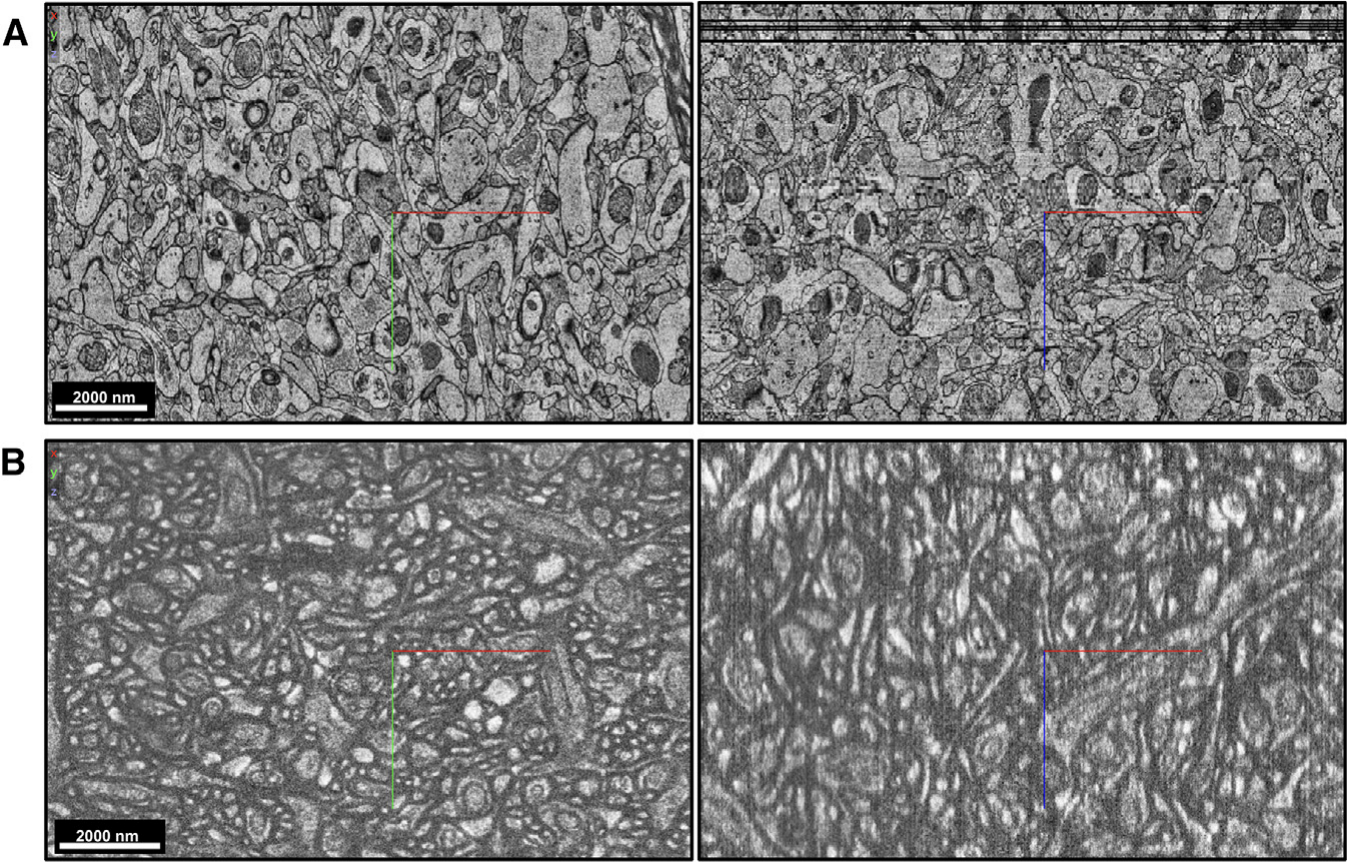
Comparison of example EM and ExM images Representative snapshots from the raw image data of (A) Shapson-Coe et al.’s ssTEM dataset of 1 mm^3^ of human cortex^19^ and (B) Tavakoli et al.’s dataset in mouse cortex using ExM with confocal microscopy.^17^ Both of these datasets were traceable using semiautomated methods (automated segmentation plus manual correction of errors). Here, the x axes are displayed as red lines, y axes as green lines, and z axes as blue lines. We obtained these snapshots from the publicly available data within NeuroGlancer.

In this analysis, we both quantitatively and qualitatively compare emerging methodologies used for imaging connectomes with a focus on the capabilities needed for the mouse brain and the human brain. The details of our calculations are available in the supplemental information. While computational image processing represents an adjacent challenge for connectomics, we focus here primarily on imaging techniques. We work under the assumption that parallel image processing developments will contribute vital new capabilities in a timely manner. In general, mammalian connectomics requires microscopy techniques featuring both high resolution and speed. Electron microscopy (EM) has, thus far, been the principal imaging method utilized in connectomics research because it achieves nanoscale resolution (Figure 1A), though it suffers from long acquisition times, even with substantial investment into ameliorating the issue.^1^^,^^12^^,^^13^ Expansion microscopy (ExM) coupled with light-sheet fluorescence microscopy (ExLSFM)^14^^,^^15^^,^^16^ represents a promising alternative route that might attain similar resolution while increasing imaging speed, but this approach is still in the early stages of development compared with EM. Tavakoli et al. recently showed that it is indeed possible to perform dense neuronal reconstruction using a 16-fold ExM approach and spinning disk confocal microscopy (Figure 1B),^17^ but there does not yet exist a high-throughput pipeline for ExM connectomics. While the existing high-throughput EM pipeline is currently the most reliable near-term path to whole mouse brain acquisition, we would argue it is likely that an ExM-based pipeline may be developed in the relatively near future. It may be beneficial to perform rigorous comparative studies between EM and ExM pipelines (once ExM pipelines are available) to aid in the planning of large-scale connectomics projects. Beyond ExLSFM, ribbon scanning confocal microscopy has shown promise as an alternative fluorescence-based modality with the potential for comparable performance to LSFM.^18^ We do not discuss this method further since it only appears in a relatively small number of publications thus far and has not yet received attention in the context of connectomics. However, we suggest that studies combining ribbon scanning confocal microscopy with ExM may yield helpful information on the competitiveness of this technique as a connectomics method. As different imaging technologies will offer complementary capabilities for connectomics, we suggest considering carefully how to best capitalize on the advantages of each of them.

In order to facilitate broad comparisons across different modalities, our analysis makes a number of simplifying assumptions. Firstly, we assume that voxel size is roughly proportional to resolution because many key studies do not report actual resolution (e.g., as measured by the Fourier shell correlation or similar). Thus, we are able to use voxel size as a common metric of comparison. Since voxel size is often not directly proportional to resolution, these numbers should be thought of as best-case scenarios. We furthermore assume that an isotropic voxel size of less than 18 nm or a non-isotropic voxel size of less than 13 × 13 × 46 nm (<13 nm lateral, <46 nm axial) has the potential for connectomic traceability (in the case of ExM, this is the effective voxel size). Several groups have used similar voxel sizes and demonstrated success with connectomic tracing.^20^^,^^21^^,^^22^ Nonetheless, it is important to realize that traceability is largely dependent on factors such as signal-to-noise ratio, contrast, the uniformity of the sample’s staining and/or expansion, the integrity of the sample’s ultrastructure, and aspects of the tracing algorithms used. In EM, there is also the problem of losing or damaging a fraction of the ultrathin slices, breaking the continuity of neuronal processes.^23^ We generally assume that samples have been prepared with sufficient precision to minimize damage, noise, staining issues, and section loss, though we do consider that baseline levels of these factors can delineate hard limits to achievable image quality. In the section on ExLSFM, we speculatively combine a state-of-the-art technique called pan-ExM-t (pan-ExM of tissue; which can reach 24-fold expansion)^15^ with light-sheet microscopes from other publications. An exception to this is our discussion of Wang et al.’s “volumetric imaging of biological specimens via photochemical sectioning” (VIPS) method, where our speculation instead involves standard 4.5-fold expansion^24^ to reach the assumed effective voxel size for nanoscale reconstruction since their microscope’s actual voxel size was quite small. We choose light-sheet microscopes, which could hypothetically achieve the aforementioned voxel sizes if their actual voxel sizes were divided by an expansion factor of 24 or 4.5. We assume that the expansion process introduces minimal distortions and minimal defects, as well as that the fluorescent labeling is of sufficient quality to allow the direct application of this expansion factor. It should be noted that this assumption is not always true: ExM can introduce long-range distortions, and expanded gels may experience damage during handling. Inconsistent demarcation of cellular boundaries is also an issue, though pan-ExM-t^15^ and new membrane labeling methods^17^^,^^25^ have substantially improved this situation. Since expanded mammalian brains may be too large for imaging as intact objects (e.g., a 24-fold expanded human brain could be over 3.5 m in length), they will need to be sectioned either before or after expansion. As will be discussed in more depth later, difficulties in the sectioning of expanded tissues could cause the loss of important information as well. While we suggest that all of these issues might be conquered by sufficient optimization efforts, they should still certainly be kept in mind when comparing the imaging modalities.

## An aside on computational constraints

Though not the central focus of this analysis, computational processing of image data may also prove a limiting factor for connectomics. Some potential areas of difficulty include the transmission bandwidth of image data moving from the detector to a storage device, the massive amount of computer memory needed to store the raw image data of whole mammalian brains, compute time for stitching and aligning images, and compute time for segmentation and synapse identification.^1^^,^^26^ Fully automated segmentation algorithms will furthermore be necessary for tracing the neurons and glia of whole mammalian brains and this problem has not yet been solved.^1^ Contributions across many fields of research will be important to overcome these challenges. Nonetheless, we are optimistic that successes in data acquisition may drive enough interest in connectomics that the necessary computational methods will be developed.

Data storage capacity represents a particular challenge for whole-brain connectomics (note that this is distinct from RAM). Assuming isotropic voxels with dimensions of 18 × 18 × 18 nm and a memory requirement of 1 byte per voxel,^27^ the raw data for a mouse brain would necessitate 85.7 petabytes and the raw data for a human brain would necessitate 205.7 exabytes of storage. As of 2023, magnetic hard disk storage cost is about $11 per terabyte,^28^ which would equate to $942,700 to store the raw image data of a mouse brain or $2,262,700,000 to store the raw image data of a human brain. Mouse brain raw image data storage may thus currently be feasible, though the raw image data storage for a human brain will likely necessitate further advances in storage technology. However, historical trends^28^ on data storage costs remain encouraging since consistent exponential decreases have been observed for several decades. Emerging technologies such as those that use femtosecond lasers to write data into quartz glass (as seen in Microsoft’s Project Silica) might offer substantially greater storage capabilities than existing magnetic storage methods.^29^ Further savings should accrue through data compression as the raw image data are processed and converted into connectome data. Indeed, a recent preprint by Li et al. demonstrated a new variational autoencoder algorithm that achieved up to 128-fold compression of EM data without significantly worsening the success of subsequent segmentation.^30^ Importantly, data compression within a high-throughput pipeline will probably take place in real time as the data are collected, which will circumvent the need to store all raw uncompressed data at once. It should also be noted that data transmission during image acquisition will likely involve parallel transmission from each microscope directly into an onsite cluster, perhaps mediated by extremely high-bandwidth hardware technology like InfiniBand.^31^ Cloud storage might also be an option, but this could present obstacles due to generally lower bandwidth and lesser reliability.^32^ Advances in hardware and software seem well on their way to handling whole mouse brain image data and may reach the point of handling whole human brain image data within the next several decades.

Image processing is another major challenge for connectomics and currently represents an even larger bottleneck than image acquisition since sufficiently accurate fully automated tracing of EM data is not yet possible^1^^,^^33^ and ExLSFM still needs to mature before such issues can be addressed thoroughly.^17^ Additionally, the amount of RAM necessary to efficiently run processing software on massive image datasets may be extremely high. It remains an open question as to the speed at which automated tracing software could segment image data, even assuming the construction of a powerful high-performance computing (HPC) center built with the singular goal of reconstructing a mouse or human brain. It will likely be critical to leverage both application-specific hardware and innovative new tracing software strategies. As a recent example of progress in tracing software, a method by Schmidt et al. called RoboEM has demonstrated the potential for more accurate automated error correction on automated connectomic segmentations.^33^ By comparing how much money was necessary for annotating past EM datasets, Schmidt et al. estimated RoboEM’s computational monetary cost at 400 times less than the monetary cost of paying for manual annotation of equivalent accuracy. (Though both RoboEM and the manual annotators still made some mistakes in annotating the tested dataset, the frequency of mistakes made was roughly equivalent.) Furthermore, RoboEM performed 3.5-fold better than state-of-the-art flood-filling networks in resolving split errors. As we will discuss more later, ExLSFM image processing requirements might drastically decrease through the use of multicolor fluorescent barcodes as labels for each neuron.^14^^,^^34^^,^^35^ This might provide a path forward for ExLSFM connectome image processing automation. Connectomics image processing is an area that still needs further maturation.

## EM

**Figure 2:**
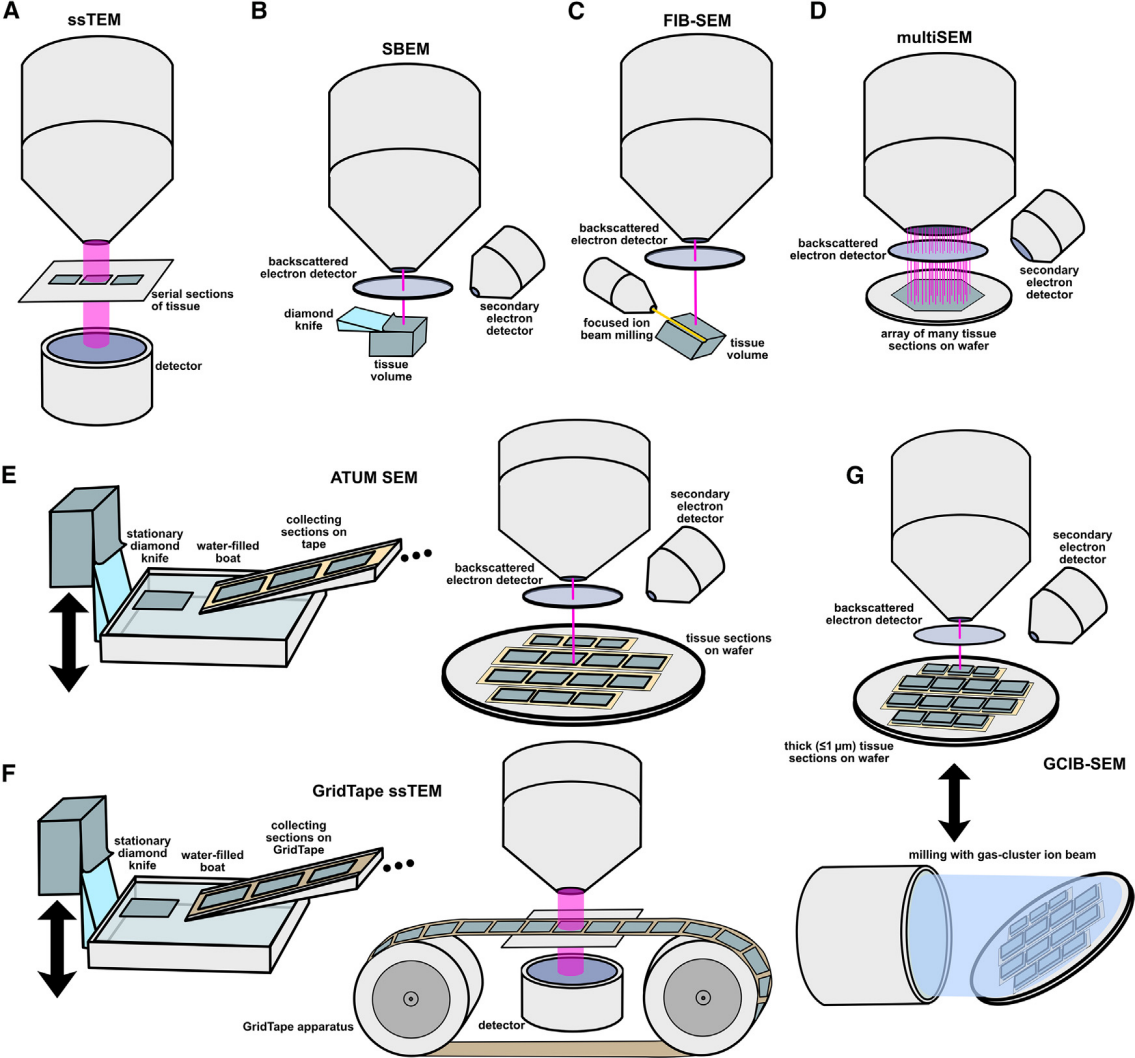
EM techniques Depictions of the mechanisms of (A) serial-section transmission electron microscopy (ssTEM), (B) serial block-face scanning electron microscopy (SBEM), (C) focused ion beam SEM (FIB-SEM), (D) multibeam SEM (multiSEM), (E) automated tape-collecting ultramicrotomy SEM (ATUM-SEM), (F) GridTape ssTEM, and (G) gas cluster ion beam SEM (GCIB-SEM). For more details on these technologies, their use in connectomics, and how they have been modified for improved throughput, see excellent reviews by Titze et al.^38^ and Peddie et al.^41^

EM represents a central method used in connectomics studies and has, so far, been the only technique that has facilitated computational reconstruction of complete whole-brain connectomes (*C. elegans*, *D. melanogaster*, and *P. dumerilii*).^3^^,^^4^^,^^5^^,^^8^^,^^36^ It involves sectioning (or milling) a sample into very thin slices and sequentially imaging each slice with an electron microscope. The most commonly used EM methods in connectomics are serial-section transmission EM (ssTEM) (Figure 2A), serial block-face EM (SBEM) (Figure 2B), and focused ion beam scanning EM (FIB-SEM) (Figure 2C).^37^ Extensive engineering efforts have gone toward increasing the throughput of these core techniques. Some important advances in the area include multibeam SEM (multiSEM) (Figure 2D),^27^ automated tape-collecting ultramicrotomy SEM (ATUM-SEM)^38^ (Figure 2E), GridTape ssTEM^39^ (Figure 2F), and gas cluster ion beam SEM (GCIB-SEM)^40^ (Figure 2G). These approaches have shown success for small volumes (i.e., up to the 1 mm^3^ scale) and may be amenable to parallelization for higher throughput, albeit at very high monetary costs. Indeed, some investigators have suggested industrial facilities with hundreds of electron microscopes working in parallel to map entire mammalian brains.^40^ Constructing and maintaining such facilities would probably cost hundreds of millions to billions of dollars, so further increases in the throughput of individual instruments may still be necessary. Nonetheless, EM brings a long history of connectomics successes, and scaling it up for the mouse brain connectome has been suggested as feasible within the coming decade.^2^

How fast are current EM methods for imaging brain tissue? (Table 1) In a landmark study by Yin et al., a 1 mm^3^ volume of brain tissue was imaged in 6 months (4 × 4 × 40 nm voxels) using a system consisting of 6 parallelized and fully automated ssTEM microscopes.^13^ The authors built this system by incorporating several custom hardware modifications into commercially available JEOL 1200EXII 120 kV TEMs as well, as by developing a software framework to facilitate a consistent automation process. Each microscope cost about $125,000, and the hardware modifications on each added a further $125,000 to the cost ($250,000 per customized microscope). As such, the total cost of the system of 6 microscopes was about $1.5 M. While these microscopes were purchased secondhand and thus were somewhat less expensive than brand-new instruments would have been, economies of scale may act to counterbalance the price of such microscopes in the future. Another example of a 1 mm^3^ acquisition comes from Shapson-Coe et al., who used ATUM to section the tissue and a multiSEM instrument (4 × 4 × 33 nm voxels) to image the tissue over the course of 326 days (10.516 months).^19^^,^^42^ The price of the multiSEM instrument was about $4 M for the version used by Shapson-Coe et al., which utilized 61 beams.^19^^,^^39^^,^^42^ It should be noted that an even faster 91-beam multiSEM is also now commercially available. These investigations represent the first steps into millimeter-scale EM connectomics and will serve as a foundation for future advances in the field.

**Table 1:** Comparison of parameters from key publications and preprints involving high-throughput EM methods for connectomics

Study	Yin et al.^13^	Shapson-Coe et al.^19^	Zheng et al.^43^
Imaging speed per microscope (mm^3^/month)	0.0278	0.0951	0.3 (projected)
Cost per microscope ($)	250,000	4 M	500,000
Voxel size (nm)	4 × 4 × 40	4 × 4 × 33	3.6 × 3.6 × 45
Time to image mouse brain per 1 microscope (years)	1,500	438	139.6
Time to image human brain per 1 microscope (years)	3.6 M	1.05 M	333,333
Time to image mouse brain with $100 M worth of microscopes	3.75 years with 400 microscopes	17.5 years with 25 microscopes	0.69 years with 200 microscopes
Time to image human brain with $100 M worth of microscopes	9,000 years with 400 microscopes	42,061 years with 25 microscopes	1,667 years with 200 microscopes

EM throughput may continue to grow over time as new technologies are developed. A promising direction employs the techniques of broad ion beam SEM (BIB-SEM) and mSTEM and combines them to make a method called BIB-mSTEM.^44^ This involves cutting thick sections of around 100–10,000 nm, imaging them via mSTEM, milling via BIB, and computationally stitching reconstructions of the thick sections. Thick sections are much easier to handle and cause less difficulty in computational reconstruction downstream than ultrathin 30-40 nm sections, so the technique has the potential to accelerate connectomics workflows by circumventing the slowdowns associated with ultrathin sectioning difficulties. Zheng et al. also greatly improved a technique called beam deflection TEM (bdTEM), which has been shown to substantially increases the throughput relative to the Yin et al. study described earlier.^43^ Beam deflection allows scanning of the electron beam over nine image tiles without moving the stage, thus eliminating eight out of nine stage movements and speeding up imaging overall. With the same type of camera, the bdTEM microscopes can each image tissue (3.6 × 3.6 × 45 nm voxels) much faster than those of the Yin et al. study. The authors estimate that if the four microscopes were to run at 65% uptime, then they could together image 1 mm^3^ in 37 days. Also, retrofitted from JEOL 1200EXII 120 kV TEMs, each of the custom bdTEMs cost about $500,000. Four of these were constructed at the Princeton facility described by Zheng et al. (about $2 M total cost). Future innovations have the potential to continue improving the throughput of EM instruments.

Although we assume in our calculations that EM sample preparation difficulties can be overcome during the design of high-throughput imaging pipelines, this part of the process remains challenging. Serial sections can suffer from inconsistent staining, warping, folding, shrinkage, and ultrastructural damage.^41^^,^^45^ Problems with staining have been greatly ameliorated by improved *en bloc* staining techniques where large tissue volumes and even whole mouse brains can be consistently penetrated by the reagents.^46^ To accelerate sectioning and improve the quality and consistency of the resulting sections, the aforementioned ATUM was developed.^47^ ATUM originally used electron-opaque support tape, limiting its use to SEM devices.^48^ More recently, an approach known as GridTape has made ATUM compatible with TEM.^39^ GridTape utilizes an electron lucent type of support tape for ATUM. It thus combines automated serial sectioning onto a TEM-compatible tape substrate with automated high-throughput imaging, creating a fully automated workflow for ssTEM. It has shown the capacity to collect over 4,000 sections (each roughly 1 mm^2^ in area) per day. At 40 nm thickness per section, this means that a ∼0.16 mm^3^ volume (roughly 1 mm^2^ area × 0.16 mm thickness) could be prepared in a day using one GridTape instrument. With the exact setup described in the original GridTape publication,^39^ sample preparation would still take a large amount of time (e.g., 8.56 years for one GridTape instrument working on a 500 mm^3^ mouse brain). However, alternative designs might greatly speed up this process. A wider diamond knife may allow the preparation of sections with substantially larger surface areas. For instance, reliable cutting of ATUM sections of 3 × 3 mm^2^ using a 4 mm blade have been reported, and even larger blades may further increase throughput on a single instrument.^38^ Cameras with wider field of view (FOV) are available for such purposes as well. Similar reasoning could apply to SBEM methods. FIB-SEM methods can also cause unintended damage to the tissue during ion beam milling, and the likelihood of inconsistent milling increases for wider tissue volumes since the energy of the beam decreases as it travels through tissue for long distances.^41^ However, GCIB-SEM may circumvent many of these issues by replacing the parallel milling beam with a perpendicular beam (of lower energy) to allow consistent milling across arbitrarily wide surfaces.^40^ Because of these factors, we suggest that imaging rate may still represent the main speed bottleneck in ssTEM, SBEM, and GCIB-SEM connectomics.

## LSFM with ExM

Coupling LSFM with ExM represents a promising up-and-coming approach for mammalian brain connectomics. LSFM utilizes specialized lenses to generate a thin sheet of laser light and excite fluorophores across a planar region of tissue.^49^ Alternatively, LSFM can utilize a rapid laser scanning approach to construct a sheet of light along the axis of the scan. In either case, an objective lens to collect light for the detector is placed perpendicular to the light sheet. The instrument translates the sheet (or the sample itself) along this perpendicular axis to collect a stack of images for reconstruction into a 3D volume. For most samples, LSFM requires tissue clearing to minimize the scattering of light. Fortunately, ExM clears tissue by virtue of volumetric dilution, so expanded tissues are translucent and amenable to LSFM.^50^ ExLSFM is a serendipitous union of technologies, combining increased resolution with the speed necessary to reconstruct large volumes.

Although ExLSFM has not yet reached a point where it is widely used in dense neural circuit mapping, it represents the subject of intensive research and seems on the cusp of feasibility as a tool for connectomics (Table 2). As mentioned earlier, Tavakoli et al. successfully achieved dense neuronal reconstruction of 16-fold expanded tissue imaged with a spinning disk confocal microscope.^17^ They imaged a 16-fold expanded piece of tissue (with a pre-expansion volume of 0.00095 mm^3^) over the course of 6.5 h. This is too slow for mammalian brain connectomics since 1 mm^3^ would take 6,841.9 h or 9.37 months. It is plausible that applying LSFM instead of spinning disk confocal microscopy could greatly accelerate this type of imaging. However, since their spinning disk confocal setup had much better axial and lateral resolution than is achievable by the faster types of LSFM (discussed in the following paragraphs), the expansion factor would likely need to be somewhat higher to still facilitate dense reconstruction. With respect to this point, another method by M’Saad et al. called pan-ExM-t has achieved 24-fold expansion as well as clear enough staining of proteins to delineate cellular boundaries in a fashion that somewhat resembles EM.^15^ While M’Saad et al.’s expansion technique was not used in combination with LSFM, it seems plausible that this will be a next step. Furthermore, in the case of lattice light-sheet microscopy (LLSM), a standard 4.5-fold expansion factor^24^ might be sufficient. A recently developed staining technique by Shin et al. has also shown the capacity to strongly label membranes in expanded and iteratively expanded brain tissue samples.^25^ This staining technique has a high potential to improve the traceability of brain tissue in the context of ExM. Along these lines, though volumetric signal dilution from high expansion factors sometimes necessitates longer exposure times for successful imaging, dense staining approaches like the aforementioned pan-labeling technique by M’Saad et al.^15^ and the membrane labeling method by Shin et al.^25^ have the potential to counteract such slowdowns. Iterative signal amplification methods^51^^,^^52^^,^^53^ or extremely bright plasmonic fluorophore labels^54^^,^^55^ may further improve staining quality in expanded tissues and thus ensure that exposure requirements do not substantially slow imaging. As will be discussed subsequently, certain LSFM setups can image at sufficiently small voxel sizes such that 24-fold expansion or even 4.5-fold expansion, perhaps along with membrane labeling, have the potential to produce EM-like resolution.

**Table 2:** Comparison of parameters from key publications and preprints involving high-throughput LSFM methods as hypothetically combined with either standard 4.5-fold expansion or 24-fold pan-ExM-t expansion as described by M’Saad et al.’s preprint and also possibly with the membrane stain from Shin et al.’s preprint.

Hypothetical combined study	Wang et al.^56^ with 4.5-fold expansion	Chakraborty et al.^57^ with 24-fold expansion^15^	Prince et al.^58^ with 24-fold expansion^15^
Actual imaging speed per microscope (mm^3^/month)	802	2,536	3,707
Cost per microscope ($)	300,000	120,000	104,000
Effective voxel size (nm) after expansion	12.4 × 12.4 × 30	17.7 × 17.7 × 17.7	17.7 × 17.7 × 17.7
Time to image expanded mouse brain per 1 microscope (years)	4.7	227	155
Time to image expanded human brain per 1 microscope (years)	11,363	545,000	373,000
Time to image expanded mouse brain with $100 M worth of microscopes	0.014 years with 333 microscopes	0.273 years with 833 microscopes	0.162 years with 962 microscopes
Time to image expanded human brain with $100 M worth of microscopes	34 years with 333 microscopes	654 years with 833 microscopes	388 years with 962 microscopes

**Figure 3:**
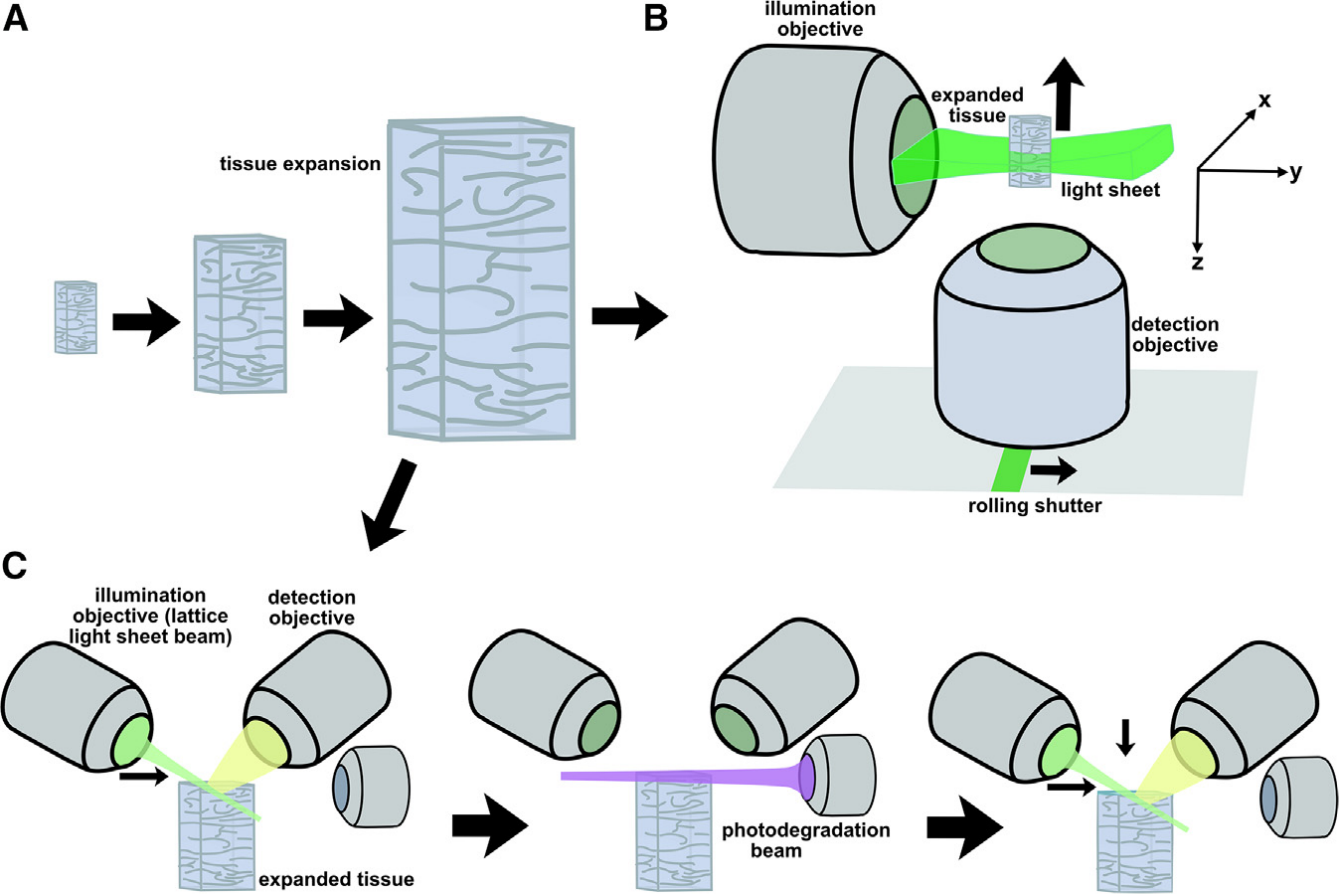
ExM techniques (A) Tissue expansion physically enlarges tissue after infusion with a swellable hydrogel and the addition of purified water. More details on expansion microscopy (ExM) can be found in excellent reviews by Truckenbrodt^50^ and Wen et al.^65^ (B) Expansion light-sheet fluorescence microscopy (ExLSFM) in an axially swept light-sheet microscopy (ASLM) configuration. (C) Expansion lattice light-sheet microscopy (ExLLSM) in a “volumetric imaging of biological specimens via photochemical sectioning” (VIPS) configuration. More details on LSFM can be found in an excellent review by Stelzer et al.^49^

What kinds of ExLSFM will be useful for imaging mammalian connectomes? (Figures 3A–3C) LLSM has received a great deal of attention,^59^^,^^60^^,^^61^ but it usually suffers from extremely small FOVs and working distance limitations, which may make it unsuitable for whole-brain mammalian connectomics, especially in the context of expanded samples.^62^^,^^63^ (Small FOVs vastly slow imaging due to volumetric scaling.) However, Wang et al. developed a promising workaround in a recent study on ExM with LLSM (ExLLSM): a photodegradable hydrogel and a UV light sheet for photochemical sectioning (Figure 3C).^56^ In this way, large tissue volumes were rapidly reconstructed through sequential ExLLSM imaging followed by controlled photodegradation of the imaged layers, effectively extending the “working distance” of the ExLLSM. They named this method VIPS. Wang et al. demonstrated the potential of their technology by imaging a mouse olfactory bulb of a 9.4 × 6.4 × 4.3 mm (258.7 mm^3^) volume after 2-fold expansion in 10 days at an actual voxel size of 56 × 56 × 135 nm (28 × 28 × 67.5 nm effective voxel size post-expansion). Although successful in reconstructing axons, this was insufficient for the reconstruction of some dendrites and synapses. As such, we will assume that 4.5-fold expansion^64^ would be sufficient for full nanoscale connectome reconstruction since it reaches a 12.4 × 12.4 × 30 nm non-isotropic voxel size (see the introduction for more details on our assumptions). With 4.5-fold expansion instead of 2-fold expansion, the same volume of 258.7 mm^3^ should take about 113.9 days to image (since the volume would be about 11.39 times larger). At this imaging speed, VIPS has promise for whole-brain mammalian connectomics, particularly if the method can be improved further. Another valuable approach may come in the form of axially swept light-sheet microscopy (ASLM) (Figure 3B). In particular, Chakraborty et al. demonstrated cleared-tissue ASLM (ctASLM), which was able to image 1 mm^3^ volumes at a 425 nm isotropic step size (voxel size) over the course of 17.6 min.^57^ If coupled with 24-fold expansion from pan-ExM-t, this would result in effective 17.7 nm isotropic voxels and would take 13,824 times longer to image (about 169 days) due to the larger volume. With an extension of the ctASLM design called signal improved ultrafast light-sheet microscopy (SIFT), Prince et al. were able to improve the imaging speed to roughly 1 mm^3^ per 9.4 min while maintaining 425 nm voxels.^58^ Once again, if coupled with 24-fold expansion from pan-ExM-t, this would result in effective 17.7 nm isotropic voxels and would once again take 13,824 times longer to image (about 90 days) due to the volume increase. It seems that the dawn of scalable ExLSFM connectomics may arrive soon given the ongoing convergence of rapidly advancing ExM and LSFM technologies.

We should also mention a highly publicized advance wherein a recent Allen Institute effort constructed an LSFM microscope called ExA-SPIM (expansion-assisted selective plane illumination microscopy), which is specially designed to rapidly image large volumes of expanded tissue at moderate voxel sizes of, at best, 1,000 × 1,000 × 2,500 nm.^66^ Though the ExA-SPIM is very fast due to its massive FOV and working distance and it can reach an imaging rate of 50 cm^3^ per day, even 24-fold expansion would only give effective voxel size of 41.67 × 41.67 × 104.17 nm. This is likely insufficient to achieve the resolution necessary for nanoscale connectomics. Because of this drawback, we will not include ExA-SPIM in our tabular comparison of ExLSFM methods for whole-brain mammalian connectomics.

Barcoding neurons by expressing unique mixtures of fluorophores or using multi-round imaging of sequences of distinct fluorophores has been proposed as a way to mitigate the need for extremely high resolution in connectomics.^14^^,^^34^^,^^35^ The feasibility of dense connectomics through a combination of Brainbow and sequential barcode methods has been supported by computational modeling on simulated data.^14^^,^^34^ However, the literature is lacking in quantitative metrics as to exactly how much these contrast mechanisms might mitigate the need for high resolution. We suggest prioritizing further quantitative investigations exploring the degree to which color contrast and barcodes may allow for connectomics imaging at coarser voxel size. It should also be noted that sequential barcodes multiply the acquisition time since they generally necessitate multiple rounds of imaging, though this is, fortunately, a linear increase rather than an exponential one. Regardless of its effect on imaging time, adding color contrast and neuronal barcodes will likely improve segmentation accuracy, particularly since existing single-color datasets are still subject to time-consuming manual proofreading steps.^4^^,^^19^

While we largely assume in our calculations that ExLSFM sample preparation difficulties can be overcome over the course of the design of high-throughput imaging pipelines, this part of the process may present unique challenges as well. In particular, there has been little work toward developing reliable strategies for either pre-expansion or post-expansion tissue sectioning. As mentioned in the introduction, the large size of a whole expanded brain will necessitate sectioning to fit within LSFM FOVs, which could destroy parts of the tissue. Furthermore, a way to hold the tissue steady during slicing will be needed to ensure clean cuts. But in the pre-expansion case, traditional forms of embedding may prove problematic since they could preclude subsequent expansion. That said, it is conceivable that one might develop a sturdy yet, under certain conditions, removable embedding medium to facilitate pre-expansion sectioning. For post-expansion sectioning, one could embed the expanded brain tissue, but the physical properties of the expansion gel might make clean cuts difficult to achieve. Laser-based cutting methods may be useable in this case since, unlike a microtome blade, they would not be prone to problems arising from the gel’s mechanical elasticity. The fine details of the neurites in expanded tissue would be physically larger; therefore, the part of the gel ablated by the laser may contain fewer cellular features than if the laser had been applied to a non-expanded sample. Indeed, the techniques of the earlier mentioned VIPS approach^56^ might be adapted for this purpose, though they would need optimization to slice tissue rather than fully ablate it. Some degree of work remains to make these possibilities a reality. We argue that once sectioning methods are established, they will contribute relatively little to the overall costs of connectome projects because a vastly smaller number of cuts will need to be made in each brain compared to the number of cuts needed for EM. Nonetheless, efficacious sectioning methods will remain crucially important to the success of whole-brain ExLSFM connectomics efforts.

## Discussion

Making comparisons across the landscape of connectome imaging technologies is difficult because distinct methods involve different technical challenges. Furthermore, some degree of extrapolation is necessary to account for the rapid development of emerging but not yet fully tested imaging pipelines. Nonetheless, careful examination of existing and emerging technologies can yield useful insights (Table 3). EM and ExLSFM have their own unique pros and cons (Table 4). EM benefits from a long history of technology development for connectomics applications and has a strong existing infrastructure for high-throughput connectomics pipelines. ExLSFM represents an emerging modality that has only recently been considered for connectomics, yet it has a speed advantage over EM and opens doors to using multiplexed fluorescent markers for simultaneously probing the brain’s molecular information.^14^ Each of these technologies deserves consideration in the context of mammalian connectomics, though developing multiple imaging modalities in parallel will likely prove the best route in the end.

**Table 3:** Direct comparisons of important characteristics of leading EM and ExLSFM approaches

	EM	ExLSFM
Time to image mouse brain with $100 M worth of fastest reported instruments	0.69 years with 200 bdTEM instruments at voxel size of 3.6 × 3.6 × 45 nm^43^	0.014 years with 333 VIPS ExLLSM instruments at effective voxel size of 12.4 × 12.4 × 30 nm^56^
Time to image human brain with $100 M worth of fastest reported instruments	1,667 years with 200 bdTEM instruments at voxel size of 3.6 × 3.6 × 45 nm^43^	34 years with 333 VIPS ExLLSM instruments at effective voxel size of 12.4 × 12.4 × 30 nm^56^
Sample preparation time and challenges	a single ATUM instrument with a 4 mm blade may section around 3 × 3 × 0.16 mm/day,^38^^,^^39^ so 200 ATUMs could section a whole mouse brain in 1.74 days or a whole human brain in 11.42 years.	ExM protocols on their own often take <1 week^15^^,^^67^; however, brains will likely need to be cut into millimeter-scale or centimeter-scale blocks, and there is currently no method for properly sectioning pre-expansion or post-expansion tissues
Raw data storage requirements	∼85.7 petabytes (cost of roughly $942,700) for mouse brain raw data and ∼205.7 exabytes (cost of roughly $2,262,700,000) for human brain raw data^28^	identical raw data storage requirements *unless* fluorescent color contrast methods (e.g., Brainbow^68^) or fluorescent barcoding^14^^,^^34^ enable acquisition of larger voxels without loss of traceability; it should be noted that the number of color channels scales linearly, while larger voxels could exponentially decrease necessary data storage amounts
Data processing requirements	much work remains to develop fully automated tracing algorithms; as there are no existing examples of fully automated tracing software, we cannot estimate compute requirements; advances in application-specific hardware may mitigate HPC costs	molecular labels in the form of fluorescent color contrast or fluorescent barcoding might help partially mollify the obstacles of automated tracing, but these approaches are still in their infancy; additionally, alignment of multiple expanded volumes (after sectioning) may present new challenges
Feasibility of mouse connectome	an EM mouse brain connectome project is feasible in the next decade or so if the fully automated tracing problem can be solved and sufficient funding is available to pay for microscopes, data storage and processing, labor costs, etc. (on the order of at least several hundred million dollars)^1^^,^^2^	an ExLSFM mouse brain connectome project is feasible in the next decade or so if ExM sample preparation and imaging pipelines mature; lack of approaches for sectioning expanded tissue volumes and reconstructing across sections hampers progress for now
Feasibility of human connectome	EM-based reconstruction of a human connectome is unlikely to occur for a very long time; alternative technological paradigms like X-ray nanotomography^69^^,^^70^^,^^71^ should be explored prior to making massive investments into a human connectome project	an ExLFSM human brain connectome project might be feasible in ∼25–50 years assuming further technological advances in both ExM and LSFM, but this remains highly uncertain; new technological paradigms such as X-ray nanotomography^69^^,^^70^^,^^71^ should be explored before making enormous investments into a human connectome project

**Table 4:** Brief overview of some advantages and some disadvantages of EM and ExLSFM

	Advantages	Disadvantages
EM	• well-established technology • track record for invertebrate connectomes • high resolution • established membrane staining methods • can reimage desired subvolumes at higher magnifications to uncover more details	• long acquisition times even with advances • involves lots of destructive slicing or milling steps • not easily compatible with molecular labeling • multiSEM instruments are very expensive
ExLSFM	• much higher speed • compatible with molecular labeling • compatible with barcoding • less slicing needed	• less well-established technology • lack of connectomics track record • less well-established membrane staining methods • imperfect expansion might damage cellular features

Imaging mammalian connectomes represents a grand challenge. Despite this, new enabling technologies are rapidly maturing. *C. elegans* and *Drosophila* connectomes have already transformed the practice of neurobiology and led to a wide variety of advances in the field. Coupled with computational modeling, mammalian connectomes could provide unprecedented insights into cognition, emotion, and brain disease. They may inform the design of AI, robotics, and brain-computer interfaces.^72^ Advanced imaging technologies are drawing mouse connectomes within reach. Mapping the human connectome remains a more distant goal, yet paradigm-shifting advances, such as synchrotron X-ray nanotomography methods^69^^,^^70^^,^^71^ and/or massive parallelization, may still make that a feasible goal sometime in the future. We anticipate that mammalian connectomes will facilitate dramatically transformative insights, improving biomedicine and perhaps even helping us better understand what it means to be human.

## Acknowledgments

We thank Dr. Anders Sandberg for engaging with us in helpful discussions that improved this paper.

## Declaration of interests

The authors declare no competing interests.

## Supplemental information

Data S1. Supplemental calculations

Document S1. Article plus supplemental information

## References

[1] Motta A., Schurr M., Staffler B., Helmstaedter M. (2019). Big data in nanoscale connectomics, and the greed for training labels. Curr. Opin. Neurobiol..

[2] Abbott L.F., Bock D.D., Callaway E.M., Denk W., Dulac C., Fairhall A.L., Fiete I., Harris K.M., Helmstaedter M., Jain V. (2020). The Mind of a Mouse. Cell.

[3] Winding M., Pedigo B.D., Barnes C.L., Patsolic H.G., Park Y., Kazimiers T., Fushiki A., Andrade I.V., Khandelwal A., Valdes-Aleman J. (2023). The connectome of an insect brain. Science.

[4] Dorkenwald S., Matsliah A., Sterling A.R., Schlegel P., Yu S.C., McKellar C.E., Lin A., Costa M., Eichler K., Yin Y. (2024). Neuronal wiring diagram of an adult brain. Nature.

[5] Schlegel P., Yin Y., Bates A.S., Dorkenwald S., Eichler K., Brooks P., Han D.S., Gkantia M., Dos Santos M., Munnelly E.J. (2024). Whole-brain annotation and multi-connectome cell typing of Drosophila. Nature.

[6] Takemura S.y., Hayworth K.J., Huang G.B., Januszewski M., Lu Z., Marin E.C., Preibisch S., Xu C.S., Bogovic J., Champion A.S. (2024). A Connectome of the Male Drosophila Ventral Nerve Cord. Elife.

[7] Azevedo A., Lesser E., Phelps J.S., Mark B., Elabbady L., Kuroda S., Sustar A., Moussa A., Khandelwal A., Dallmann C.J. (2024). Connectomic reconstruction of a female Drosophila ventral nerve cord. Nature.

[8] Verasztó C., Jasek S., Gühmann M., Bezares-Calderón L.A., Williams E.A., Shahidi R., Jékely G. (2024). Whole-body connectome of a segmented annelid larva. bioRxiv.

[9] White J.G., Southgate E., Thomson J.N., Brenner S. (1986). The structure of the nervous system of the nematode Caenorhabditis elegans. Philos. Trans. R Soc. L. B Biol. Sci..

[10] Cosgrove K.P., Mazure C.M., Staley J.K. (2007). Evolving Knowledge of Sex Differences in Brain Structure, Function, and Chemistry. Biol. Psychiatr..

[11] Allen J.S., Damasio H., Grabowski T.J. (2002). Normal neuroanatomical variation in the human brain: An MRI-volumetric study. Am. J. Phys. Anthropol..

[12] Mikula S. (2016). Progress Towards Mammalian Whole-Brain Cellular Connectomics. Front. Neuroanat..

[13] Yin W., Brittain D., Borseth J., Scott M.E., Williams D., Perkins J., Own C.S., Murfitt M., Torres R.M., Kapner D. (2020). A petascale automated imaging pipeline for mapping neuronal circuits with high-throughput transmission electron microscopy. Nat. Commun..

[14] Yoon Y.-G., Dai P., Wohlwend J., Chang J.B., Marblestone A.H., Boyden E.S. (2017). Feasibility of 3D Reconstruction of Neural Morphology Using Expansion Microscopy and Barcode-Guided Agglomeration. Front. Comput. Neurosci..

[15] M’Saad O., Bewersdorf J. (2022). All-optical visualization of specific molecules in the ultrastructural context of brain tissue. bioRxiv.

[16] Lillvis J.L., Otsuna H., Ding X., Pisarev I., Kawase T., Colonell J., Rokicki K., Goina C., Gao R., Hu A. (2022). Rapid reconstruction of neural circuits using tissue expansion and light sheet microscopy. Elife.

[17] Tavakoli M.R., Lyudchik J., Januszewski M., Vistunou V., Agudelo N., Vorlaufer J., Sommer C., Kreuzinger C., Oliveira B., Cenameri A. (2024). Light-microscopy based dense connectomic reconstruction of mammalian brain tissue. bioRxiv.

[18] Watson A.M., Rose A.H., Gibson G.A., Gardner C.L., Sun C., Reed D.S., Lam L.K.M., St Croix C.M., Strick P.L., Klimstra W.B., Watkins S.C. (2017). Ribbon scanning confocal for high-speed high-resolution volume imaging of brain. PLoS One.

[19] Shapson-Coe A., Januszewski M., Berger D.R., Pope A., Wu Y., Blakely T., Schalek R.L., Li P.H., Wang S., Maitin-Shepard J. (2024). A petavoxel fragment of human cerebral cortex reconstructed at nanoscale resolution. Science.

[20] Motta A., Berning M., Boergens K.M., Staffler B., Beining M., Loomba S., Hennig P., Wissler H., Helmstaedter M. (2019). Dense connectomic reconstruction in layer 4 of the somatosensory cortex. Science.

[21] Schmidt H., Gour A., Straehle J., Boergens K.M., Brecht M., Helmstaedter M. (2017). Axonal synapse sorting in medial entorhinal cortex. Nature.

[22] Xu C.S., Pang S., Hayworth K.J., Hess H.F. (2019). Enabling FIB-SEM Systems for Large Volume Connectomics and Cell Biology. bioRxiv.

[23] Lee K., Turner N., Macrina T., Wu J., Lu R., Seung H.S. (2019). Convolutional nets for reconstructing neural circuits from brain images acquired by serial section electron microscopy. Curr. Opin. Neurobiol..

[24] Asano S.M., Gao R., Wassie A.T., Tillberg P.W., Chen F., Boyden E.S. (2018). Expansion Microscopy: Protocols for Imaging Proteins and RNA in Cells and Tissues. Curr. Protoc. Cell Biol..

[25] Shin T.W., Wang H., Zhang C., An B., Lu Y., Zhang E., Lu X., Karagiannis E.D., Kang J.S., Emenari A. (2024). Dense, Continuous Membrane Labeling and Expansion Microscopy Visualization of Ultrastructure in Tissues. bioRxiv.

[26] Beyer J., Troidl J., Boorboor S., Hadwiger M., Kaufman A., Pfister H. (2022). A Survey of Visualization and Analysis in High-Resolution Connectomics. Comput. Graph. Forum.

[27] Eberle A.L., Zeidler D. (2018). Multi-Beam Scanning Electron Microscopy for High-Throughput Imaging in Connectomics Research. Front. Neuroanat..

[28] Our World in Data: Historical price of computer memory and storage. https://ourworldindata.org/grapher/historical-cost-of-computer-memory-and-storage (2024).

[29] Anderson P., Aranas E.B., Assaf Y., Behrendt R., Black R., Caballero M., Cameron P., Canakci B., De Carvalho T., Chatzieleftheriou A., Storan Clarke R. (2023). Proceedings of the 29th Symposium on Operating Systems Principles.

[30] Li Y., Park C.F., Xenes D., Bishop C., Berger D.R., Samuel A.D., Wester B., Lichtman J.W., Pfister H., Li W., Meirovitch Y. (2024). EM-Compressor: Electron Microscopy Image Compression in Connectomics with Variational Autoencoders. bioRxiv.

[31] Lu P.-J., Lai M.-C., Chang J.-S. (2022). A Survey of High-Performance Interconnection Networks in High-Performance Computer Systems. Electronics.

[32] Tahir A., Chen F., Khan H.U., Ming Z., Ahmad A., Nazir S., Shafiq M. (2020). A Systematic Review on Cloud Storage Mechanisms Concerning e-Healthcare Systems. Sensors.

[33] Schmidt M., Motta A., Sievers M., Helmstaedter M. (2024). RoboEM: automated 3D flight tracing for synaptic-resolution connectomics. Nat. Methods.

[34] Chen S., Loper J., Zhou P., Paninski L. (2022). Blind demixing methods for recovering dense neuronal morphology from barcode imaging data. PLoS Comput. Biol..

[35] Shen F.Y., Harrington M.M., Walker L.A., Cheng H.P.J., Boyden E.S., Cai D. (2020). Light microscopy based approach for mapping connectivity with molecular specificity. Nat. Commun..

[36] Cook S.J., Jarrell T.A., Brittin C.A., Wang Y., Bloniarz A.E., Yakovlev M.A., Nguyen K.C.Q., Tang L.T.H., Bayer E.A., Duerr J.S. (2019). Whole-animal connectomes of both Caenorhabditis elegans sexes. Nature.

[37] Kubota Y., Sohn J., Kawaguchi Y. (2018). Large volume electron microscopy and neural microcircuit analysis. Front. Neural Circ..

[38] Titze B., Genoud C. (2016). Volume scanning electron microscopy for imaging biological ultrastructure. Biol. Cell.

[39] Graham B.J., Hildebrand D.G.C., Kuan A.T., Maniates-Selvin J.T., Thomas L.A., Shanny B.L., Lee W.C.A. (2019). High-throughput transmission electron microscopy with automated serial sectioning. bioRxiv.

[40] Hayworth K.J., Peale D., Januszewski M., Knott G.W., Lu Z., Xu C.S., Hess H.F. (2020). Gas cluster ion beam SEM for imaging of large tissue samples with 10 nm isotropic resolution. Nat. Methods.

[41] Peddie C.J., Genoud C., Kreshuk A., Meechan K., Micheva K.D., Narayan K., Pape C., Parton R.G., Schieber N.L., Schwab Y. (2022). Volume electron microscopy. Nat. Rev. Methods Prim..

[42] Shapson-Coe A., Januszewski M., Berger D.R., Pope A., Wu Y., Blakely T., Schalek R.L., Li P.H., Wang S., Maitin-Shepard J. (2021). A connectomic study of a petascale fragment of human cerebral cortex. bioRxiv.

[43] Zheng Z., Own C.S., Wanner A.A., Koene R.A., Hammerschmith E.W., Silversmith W.M., Kemnitz N., Lu R., Tank D.W., Seung H.S. (2024). Fast imaging of millimeter-scale areas with beam deflection transmission electron microscopy. Nat. Commun..

[44] Kormacheva M., Kievits A., Reuteler J., Niessen M., Hoedt S.d., Khan S., Bosch C., Hoogenboom J., Schaefer A., Wanner A. (2024). BIB-mSTEM Approach for Large Scale Acquisition of Brain Tissue. Microsc. Microanal..

[45] Ohno N., Katoh M., Saitoh Y., Saitoh S., Ohno S. (2015). Three-dimensional volume imaging with electron microscopy toward connectome. Microscopy.

[46] Song K., Feng Z., Helmstaedter M. (2023). High-contrast en bloc staining of mouse whole-brain and human brain samples for EM-based connectomics. Nat. Methods.

[47] Hayworth K.J., Kasthuri N., Schalek R., Lichtman J.W. (2006). Automating the Collection of Ultrathin Serial Sections for Large Volume TEM Reconstructions. Microsc. Microanal..

[48] Briggman K.L., Bock D.D. (2012). Volume electron microscopy for neuronal circuit reconstruction. Curr. Opin. Neurobiol..

[49] Stelzer E.H.K., Strobl F., Chang B.J., Preusser F., Preibisch S., McDole K., Fiolka R. (2021). Light sheet fluorescence microscopy. Nat. Rev. Methods Primers.

[50] Truckenbrodt S. (2023). Expansion Microscopy: Super-Resolution Imaging with Hydrogels. Anal. Chem..

[51] Cho Y., Seo J., Sim Y., Chung J., Park C.E., Park C.G., Kim D., Chang J.B. (2020). FRACTAL: Signal amplification of immunofluorescence via cyclic staining of target molecules. Nanoscale.

[52] Saka S.K., Wang Y., Kishi J.Y., Zhu A., Zeng Y., Xie W., Kirli K., Yapp C., Cicconet M., Beliveau B.J. (2019). Immuno-SABER enables highly multiplexed and amplified protein imaging in tissues. Nat. Biotechnol..

[53] Wassie A.T., Zhao Y., Boyden E.S. (2019). Expansion microscopy: principles and uses in biological research. Nat. Methods.

[54] Rathi P., Gupta P., Debnath A., Baldi H., Wang Y., Gupta R., Raman B., Singamaneni S. (2023). Plasmon-Enhanced Expansion Microscopy. Nano Lett..

[55] Artur C.G., Womack T., Zhao F., Eriksen J.L., Mayerich D., Shih W.C. (2018). Plasmonic nanoparticle-based expansion microscopy with surface-enhanced Raman and dark-field spectroscopic imaging. Biomed. Opt Express.

[56] Wang W., Ruan X., Liu G., Milkie D.E., Li W., Betzig E., Upadhyayula S., Gao R. (2024). Nanoscale volumetric fluorescence imaging via photochemical sectioning. bioRxiv.

[57] Chakraborty T., Driscoll M.K., Jeffery E., Murphy M.M., Roudot P., Chang B.J., Vora S., Wong W.M., Nielson C.D., Zhang H. (2019). Light-sheet microscopy of cleared tissues with isotropic, subcellular resolution. Nat. Methods.

[58] Prince M.N.H., Garcia B., Henn C., Yi Y., Susaki E.A., Watakabe Y., Nemoto T., Lidke K.A., Zhao H., Remiro I.S. (2024). Signal improved ultra-fast light-sheet microscope for large tissue imaging. Commun. Eng..

[59] Chen B.C., Legant W.R., Wang K., Shao L., Milkie D.E., Davidson M.W., Janetopoulos C., Wu X.S., Hammer J.A., Liu Z. (2014). Lattice light-sheet microscopy: Imaging molecules to embryos at high spatiotemporal resolution. Science.

[60] Gao R., Asano S.M., Upadhyayula S., Pisarev I., Milkie D.E., Liu T.L., Singh V., Graves A., Huynh G.H., Zhao Y. (2019). Cortical column and whole-brain imaging with molecular contrast and nanoscale resolution. Science.

[61] Liu T.-L., Upadhyayula S., Milkie D.E., Singh V., Wang K., Swinburne I.A., Mosaliganti K.R., Collins Z.M., Hiscock T.W., Shea J. (2018). Observing the cell in its native state: Imaging subcellular dynamics in multicellular organisms. Science.

[62] Tsai Y.-C., Tang W.C., Low C.S.L., Liu Y.T., Wu J.S., Lee P.Y., Chen L.Q., Lin Y.L., Kanchanawong P., Gao L., Chen B.C. (2020). Rapid high resolution 3D imaging of expanded biological specimens with lattice light sheet microscopy. Methods.

[63] Shi Y., Daugird T.A., Legant W.R. (2022). A quantitative analysis of various patterns applied in lattice light sheet microscopy. Nat. Commun..

[64] Park H.-E., Choi D., Park J.S., Sim C., Park S., Kang S., Yim H., Lee M., Kim J., Pac J. (2019). Scalable and Isotropic Expansion of Tissues with Simply Tunable Expansion Ratio. Adv. Sci..

[65] Wen G., Leen V., Rohand T., Sauer M., Hofkens J. (2023). Current Progress in Expansion Microscopy: Chemical Strategies and Applications. Chem. Rev..

[66] Glaser A., Chandrashekar J., Vasquez S., Arshadi C., Ouellette N., Jiang X., Baka J., Kovacs G., Woodard M., Seshamani S. (2024). Expansion-assisted selective plane illumination microscopy for nanoscale imaging of centimeter-scale tissues. bioRxiv.

[67] Truckenbrodt S., Sommer C., Rizzoli S.O., Danzl J.G. (2019). A practical guide to optimization in X10 expansion microscopy. Nat. Protoc..

[68] Cai D., Cohen K.B., Luo T., Lichtman J.W., Sanes J.R. (2013). Improved tools for the Brainbow toolbox. Nat. Methods.

[69] Kuan A.T., Phelps J.S., Thomas L.A., Nguyen T.M., Han J., Chen C.L., Azevedo A.W., Tuthill J.C., Funke J., Cloetens P. (2020). Dense neuronal reconstruction through X-ray holographic nano-tomography. Nat. Neurosci..

[70] Stampfl A.P., Liu Z., Hu J., Sawada K., Takano H., Kohmura Y., Ishikawa T., Lim J.H., Je J.H., Low C.M. (2023). SYNAPSE: An international roadmap to large brain imaging. Phys. Rep..

[71] Bosch C., Aidukas T., Holler M., Pacureanu A., Müller E., Peddie C.J., Zhang Y., Cook P., Collinson L., Bunk O. (2024). Non-destructive X-ray tomography of brain tissue ultrastructure. bioRxiv.

[72] Collins L.T. (2019). The case for emulating insect brains using anatomical “wiring diagrams” equipped with biophysical models of neuronal activity. Biol. Cybern..

