# Supplementary material for: Comparative prospects of imaging methods for whole-brain mammalian connectomics
Source: Cell Rep Methods. 2025 Feb 18;5(2):100988. doi: 10.1016/j.crmeth.2025.100988 (PMC11955263; doi:10.1016/j.crmeth.2025.100988)
Supplement: Data S1. Supplemental calculations [file mmc1.pdf]

Cell Reports Methods, Volume *5*

Supplemental information

Comparative prospects of imaging methods for whole-brain mammalian connectomics

Logan Thrasher Collins, Todd Huffman, and Randal Koene

Supplemental calculations:

Electron microscopy:

Yin et al. (2020)[1]

Raw numbers listed in the paper:
Total acquisition time: ~6 months
Total volume: ~1 mm^3
Number of microscopes running in parallel: 6
Voxel size: 4×40×40 nm
Cost per microscope: $250,000 ($125,000 for microscope plus $125,000 for modifications)

Imaging speed per microscope (mm^3/month) calculation:
$1\ \text{mm}^3\ /\ 6\ \text{months}\ /\ 6\ \text{microscopes}\ =\ 0.0278\ \text{mm}^3/\text{month}$

Time to image mouse brain per 1 microscope calculation:
$500\ \text{mm}^3\ /\ (0.0278\ \text{mm}^3/\text{month})\ =\ 18{,}000\ \text{months}$
$18{,}000/12\ =\ 1{,}500\ \text{years}$

Time to image human brain per 1 microscope calculation:
$1{,}200{,}000\ \text{mm}^3\ /\ (0.0278\ \text{mm}^3/\text{month})\ =\ 4.3165 \times 10^7\ \text{months}$
$(4.3165 \times 10^7)/12\ =\ 3.5971 \times 10^6$ years (rounded to 3.6M years)

Time to image mouse brain with $100M of microscopes calculation:
Number of microscopes is $\$10^8/\$250{,}000\ =\ 400$ microscopes
$500\ \text{mm}^3/(400 \bullet 0.0278\ \text{mm}^3/\text{month}) = 44.964$ months
$(44.964)/12 = 3.747$ years (rounded to 3.75 years)

Time to image human brain with $100M of microscopes calculation:
Number of microscopes is $\$10^8/\$250{,}000\ =\ 400$ microscopes
$1{,}200{,}000\ \text{mm}^3/(400 \bullet 0.0278\ \text{mm}^3/\text{month}) = 1.0791 \times 10^5$ months
$(1.0791 \times 10^5)/12 = 8{,}992.8$ years (rounded to 9,000 years)

Shapson-Coe et al. (2021)[2]

Raw numbers listed in the paper (and elsewhere as noted):
Total acquisition time: 326 days
Total volume: ~1 mm^3
Number of microscopes running in parallel:
Voxel size: 4×40×33 nm
Cost per microscope: $4,000,000, see table 1 of "High-throughput transmission electron microscopy with automated serial sectioning"[3] for multibeam SEM prices.

Imaging speed per microscope (mm^3/month) calculation:
$326\ \text{days}\ /\ 31 = 10.5161\ \text{months}$
$1\ \text{mm}^3\ /\ 10.5161\ \text{months}\ /\ 1\ \text{microscopes}\ =\ 0.0951\ \text{mm}^3/\text{month}$

Time to image mouse brain per 1 microscope calculation:

$500\ \text{mm}^3\ /\ (0.0951\ \text{mm}^3/\text{month})\ =\ 5{,}257.6$ months
$5{,}257.6/12\ =\ 438.1353$ years (rounded to 438 years)

Time to image human brain per 1 microscope calculation:
$1{,}200{,}000\ \text{mm}^3\ /\ (0.0951\ \text{mm}^3/\text{month})\ =\ 1.2618 \times 10^7$ months
$(1.2618 \times 10^7)/12\ =\ 1.0515 \times 10^6$ years (rounded to 1.05×10^6 years)

Time to image mouse brain with $100M of microscopes calculation:
Number of microscopes is $\$10^8/\$4{,}000{,}000\ =\ 25$ microscopes
$500\ \text{mm}^3/(25 \bullet 0.0951\ \text{mm}^3/\text{month}) = 210.3049$ months
$(210.3049)/12 = 17.5254$ years (rounded to 17.5 years)

Time to image human brain with $100M of microscopes calculation:
Number of microscopes is $\$10^8/\$4{,}000{,}000\ =\ 25$ microscopes
$1{,}200{,}000\ \text{mm}^3/(25 \bullet 0.0951\ \text{mm}^3/\text{month}) = 5.0473 \times 10^5$ months
$(5.0473 \times 10^5)/12 = 42{,}061$ years

Zheng et al. (2021)[4]

Raw numbers listed in the paper (and elsewhere as noted):
Projected acquisition time to image 1 mm^3 with 4 microscopes in parallel: 37 days (assuming 65% uptime)
Number of microscopes running in parallel: 4
Voxel size: 3.6×3.6×45 nm
Cost per microscope: $500,000

Imaging speed per microscope (mm^3/month) calculation:
37 days $/\ 31\ =\ 1.1935$ months for $1\ \text{mm}^3$
$1.1935\ \text{months}/\text{mm}^3\ /\ 4$ microscopes $=\ 0.2984\ \text{months}/\text{mm}^3/\text{microscope}$
(rounded to $0.3\ \text{mm}^3/\text{month}$ for 1 microscope)

Time to image mouse brain per 1 microscope calculation:
$500\ \text{mm}^3\ /\ (0.3\ \text{mm}^3/\text{month})\ =\ 1{,}666.7$ months
$1{,}666.7/12\ = 139.6336$ years (rounded to 139.6 years)

Time to image human brain per 1 microscope calculation:
$1{,}200{,}000\ \text{mm}^3\ /\ (0.3\ \text{mm}^3/\text{month})\ =\ 4{,}000{,}000$ months
$4{,}000{,}000/12\ = 333{,}333.33$ years (rounded to 333,333 years)

Time to image mouse brain with $100M of microscopes calculation:
Number of microscopes is $\$10^8/\$500{,}000\ =\ 200$ microscopes
$500\ \text{mm}^3/(200 \bullet 0.3\ \text{mm}^3/\text{month}) = 8.333$ months
$(8.333)/12 = 0.6944$ years (rounded to 0.69 years)

Time to image human brain with $100M of microscopes calculation:
Number of microscopes is $\$10^8/\$500{,}000\ =\ 200$ microscopes
$1{,}200{,}000\ \text{mm}^3/(200 \bullet 0.3\ \text{mm}^3/\text{month}) = 20{,}000$ months
$(20{,}000)/12 = 1{,}666.7$ years (rounded to 1,667 years)

Expansion light-sheet fluorescence microscopy

Wang et al. (2024)

Raw numbers listed in the paper (and elsewhere as noted):
Number of microscopes running in parallel: 1
Voxel size: 56×56×135 nm
Cost per microscope: \$300,000 as found through personal correspondence with the authors.
Acquisition speed: 9.4×6.4×4.3 mm (after 2-fold expansion) per 10 days

Effective voxel size with 7-fold expansion[5] calculation:
$56 \times 56 \times 135 \text{ nm} / 4.5 = 12.44 \times 12.44 \times 30 \text{ nm voxels} = 4{,}645.6 \text{ nm}^3$ volume per voxel

Imaging speed per microscope (mm^3/month) calculation
$(9.4)(6.4)(4.3)\text{ mm}^3/(10\text{ days}) = 1 \bullet (25.87\text{ mm}^3/\text{day})(31\text{ days per month})$
$= 801.97\text{ mm}^3/\text{ month (rounded to } 802\text{ mm}^3/\text{month)}$

Time to image 4.5-fold expanded mouse brain per 1 microscope calculation:
$(500\text{ mm}^3 \bullet 4.5^3) / (801.97\text{ mm}^3/\text{month}) = 56.81\text{ months}$
$56.81/12 = 4.734$ years (rounded to 4.7 years)

Time to image 4.5-fold expanded human brain per 1 microscope calculation:
$(1{,}200{,}000\text{ mm}^3 \bullet 4.5^3) / (801.97\text{ mm}^3/\text{month}) = 136{,}350\text{ months}$
$(136{,}350)/12 = 11{,}363$ years

Time to image 4.5-fold expanded mouse brain with \$100M of microscopes calculation:
Number of microscopes is $\$10^8/\$300{,}000 = 333$ microscopes
$(500\text{ mm}^3 \bullet 4.5^3) / (333\text{ microscopes} \bullet 801.97\text{ mm}^3/\text{month}) = 0.171\text{ months}$
$0.171/12 = 0.0142$ years (rounded to 0.014 years)

Time to image 4.5-fold expanded human brain with \$100M of microscopes calculation:
Number of microscopes is $\$10^8/\$300{,}000 = 333$ microscopes
$(1{,}200{,}000\text{ mm}^3 \bullet 4.5^3) / (333\text{ microscopes} \bullet 801.97\text{ mm}^3/\text{month}) = 409.46\text{ months}$
$409.46/12 = 34.12$ years (rounded to 34 years)

Chakraborty et al. (2019)[6]

Raw numbers listed in the paper (and elsewhere as noted):
Number of microscopes running in parallel: 1
Voxel size: 425×425×425 nm, see passage in Chakraborty et al.'s Methods "*an isotropic step size of 0.425 µm and 18% overlap between stacks for faithful stitching*" and their Supplementary Table 6.
Cost per microscope: \$120,000; see Chakraborty et al.'s Supplementary Table 5.
Acquisition speed: 17.6 minutes per mm^3, see passage in Chakraborty et al.'s Methods "*Imaging a 1-mm^3 volume using ctASLM took 17.6 min*".

Effective voxel size with 24-fold expansion[7] calculation:
$425\text{ nm isotropic}/24 = 17.7\text{ nm isotropic voxels}$

Imaging speed per microscope (mm^3/month) calculation:

$1\ \mathrm{mm}^3/17.6\ \text{minutes} = 1 \bullet (44{,}640\ \text{minutes per month} / 17.6)$
$= 2{,}536.36$ (rounded to $2{,}536\ \mathrm{mm}^3/\text{month}$)

Time to image 24-fold expanded[7] mouse brain per 1 microscope calculation:
$(500\ \mathrm{mm}^3 \bullet 24^3) / (2{,}536\ \mathrm{mm}^3/\text{month}) = 2{,}725.6$ months
$2{,}725.6/12 = 227.1293$ years (rounded to 227 years)

Time to image 24-fold expanded[7] human brain per 1 microscope calculation:
$(1{,}200{,}000\ \mathrm{mm}^3 \bullet 24^3) / (2{,}536\ \mathrm{mm}^3/\text{month}) = 6.5413 \times 10^6$ months
$(6.5413 \times 10^6)/12 = 5.4511 \times 10^5$ years (rounded to 5.45×10^5 years)

Time to image 24-fold expanded[7] mouse brain with \$100M of microscopes calculation:
Number of microscopes is $\$10^8/\$120{,}000 = 833$ microscopes
$(500\ \mathrm{mm}^3 \bullet 24^3) / (833\ \text{microscopes} \bullet 2{,}536\ \mathrm{mm}^3/\text{month}) = 3.272$ months
$3.272/12 = 0.2727$ years (rounded to 0.273 years)

Time to image 24-fold expanded[7] human brain with \$100M of microscopes calculation:
Number of microscopes is $\$10^8/\$120{,}000 = 833$ microscopes
$(1{,}200{,}000\ \mathrm{mm}^3 \bullet 24^3) / (833\ \text{microscopes} \bullet 2{,}536\ \mathrm{mm}^3/\text{month}) = 7{,}852.7$ months
$7{,}852.7/12 = 654.3942$ years (rounded to 654 years)

Prince et al. (2023)[8]

Raw numbers listed in the paper (and elsewhere as noted):
Number of microscopes running in parallel: 1
Voxel size: 425×425×425 nm, see passage in Prince et al.'s paper "*The axial step size is determined by the lateral pixel size at the image space. For instance, the measured magnification of SIFT in water is 15.28x, which gives a lateral image pixel size of 0.425 µm*" and their Supplementary Table 3. Slightly better magnifications were possible in other immersion media, but we have chosen to use the value for water since expanded tissues are typically filled with pure water.
Cost per microscope: \$104,000 as found through personal correspondence with the authors.
Acquisition speed: 5.5×3.6×3.5 mm^3 / 13.91 hours, see passage in Prince et al.'s paper "*although the total pfAT of imaging 5.5 × 4.6 × 3.5 mm³ mouse hind-paw for SIFT and traditional ASLM are 7.63 and 30.55 hours respectively, the total imaging time for SIFT and traditional ASLM are 13.91 and 40.48 hours respectively*".

Effective voxel size with 24-fold expansion[7] calculation:
425 nm isotropic$/24 = 17.7$ nm isotropic voxels

Imaging speed per microscope (mm^3/month) calculation:
$(5.5)(3.6)(3.5)\ \mathrm{mm}^3/13.91\ \text{hours} = 69.3\ \mathrm{mm}^3/\ 13.91(60) = 0.083\ \mathrm{mm}^3/\text{minute}$
$= (0.083\ \mathrm{mm}^3)(44{,}640\ \text{minutes per month}) = 3{,}706.6$ (rounded to $3{,}707\ \mathrm{mm}^3/\text{month}$)

Time to image 24-fold expanded[7] mouse brain per 1 microscope calculation:
$(500\ \mathrm{mm}^3 \bullet 24^3) / (3{,}707\ \mathrm{mm}^3/\text{month}) = 1{,}864.6$ months
$1{,}864.6/12 = 155.3817$ years (rounded to 155 years)

Time to image 24-fold expanded[7] human brain per 1 microscope calculation:
$(1{,}200{,}000\ \mathrm{mm}^3 \bullet 24^3) / (3{,}707\ \mathrm{mm}^3/\text{month}) = 4.475 \times 10^6$ months
$(4.475 \times 10^6)/12 = 3.7292 \times 10^5$ years (rounded to 3.73×10^5 years)

Time to image 24-fold expanded[7] mouse brain with $100M of microscopes calculation:
Number of microscopes is $\$10^8/\$104{,}000 = 962$ microscopes
$(500\ \text{mm}^3 \bullet 24^3) / (962\ \text{microscopes} \bullet 3{,}707\ \text{mm}^3/\text{month}) = 1.9382$ months
$1.9382/12 = 0.1615$ years (rounded to 0.162 years)

Time to image 24-fold expanded[7] human brain with $100M of microscopes calculation:
Number of microscopes is $\$10^8/\$120{,}000 = 962$ microscopes
$(1{,}200{,}000\ \text{mm}^3 \bullet 24^3) / (962\ \text{microscopes} \bullet 3{,}707\ \text{mm}^3/\text{month}) = 4{,}651.8$ months
$4{,}651.8/12 = 387.6467$ years (rounded to 388 years)

Supplemental references:

1. Yin, W. *et al.* A petascale automated imaging pipeline for mapping neuronal circuits with high-throughput transmission electron microscopy. *Nat. Commun.* **11**, 4949 (2020).
2. Shapson-Coe, A. *et al.* A connectomic study of a petascale fragment of human cerebral cortex. *bioRxiv* 2021.05.29.446289 (2021) doi:10.1101/2021.05.29.446289.
3. Graham, B. J. *et al.* High-throughput transmission electron microscopy with automated serial sectioning. *bioRxiv* 657346 (2019) doi:10.1101/657346.
4. Zheng, Z. *et al.* Fast imaging of millimeter-scale areas with beam deflection transmission electron microscopy. *bioRxiv* 2022.11.23.517701 (2022) doi:10.1101/2022.11.23.517701.
5. Park, H.-E. *et al.* Scalable and Isotropic Expansion of Tissues with Simply Tunable Expansion Ratio. *Adv. Sci.* **6**, 1901673 (2019).
6. Chakraborty, T. *et al.* Light-sheet microscopy of cleared tissues with isotropic, subcellular resolution. *Nat. Methods* **16**, 1109–1113 (2019).
7. M'Saad, O. *et al.* All-optical visualization of specific molecules in the ultrastructural context of brain tissue. *bioRxiv* 2022.04.04.486901 (2022) doi:10.1101/2022.04.04.486901.
8. Prince, M. N. H. *et al.* Signal Improved ultra-Fast Light-sheet Microscope (SIFT) for large tissue imaging. *bioRxiv* 2023.05.31.543002 (2023) doi:10.1101/2023.05.31.543002.
